# Clinical proof of concept for anti-FGF2 therapy in exudative age-related macular degeneration (nAMD): phase 2 trials in treatment-naïve and anti-VEGF pretreated patients

**DOI:** 10.1038/s41433-023-02848-7

**Published:** 2023-11-30

**Authors:** Daniel S. Pereira, Raj K. Maturi, Kazumasa Akita, Vinaya Mahesh, Robert B. Bhisitkul, Toshiaki Nishihata, Eri Sakota, Yusuf Ali, Emiko Nakamura, Padma Bezwada, Yoshikazu Nakamura

**Affiliations:** 1 RIBOMIC USA Inc., Berkeley, CA USA; 2 Midwest Eye Institute, Indianapolis, IN, and Department of Ophthalmology, Indiana University School of Medicine, Indianapolis, IN USA; 3 RIBOMIC Inc., Minato-ku, Tokyo, Japan; 4 Department of Ophthalmology, University of California San Francisco, San Francisco, CA USA; 5 Institute of Medical Science, The University of Tokyo, Minato-ku, Tokyo, Japan

**Keywords:** Drug discovery, Medical research

## Abstract

**Background/Objective:**

Intravitreal injections of anti-vascular endothelial growth factor (VEGF) agents are the first-line treatment for exudative age-related macular degeneration (nAMD). Due to the limitations of these standard therapies, targeting alternative mechanisms of action may be helpful for treatment of this very common disease. Here, we investigated an anti-fibroblast growth factor-2 (FGF2) aptamer, umedaptanib pegol, a next generation therapeutic for the treatment of nAMD.

**Methods:**

Three phase 2 studies were designed. First, a multicentre, randomized, double-masked TOFU study assessed the efficacy of intravitreal injections of umedaptanib pegol monotherapy or in combination with aflibercept, compared to aflibercept monotherapy in 86 subjects with anti-VEGF pretreated nAMD. Second, 22 subjects who had exited the TOFU study received 4 monthly intravitreal injections of umedaptanib pegol (extension, RAMEN study). Third, as an investigator-sponsored trial (TEMPURA study), a single-center, open-label, 4-month study was designed to evaluate the safety and treatment efficacy of umedaptanib pegol in five naïve nAMD patients who had not received any prior anti-VEGF treatment.

**Results:**

The TOFU study demonstrated that umedaptanib pegol alone or in combination with aflibercept did not improve best-corrected visual acuity (BCVA) and central subfield thickness (CST) over aflibercept alone. However, the change in BCVA and CST at primary endpoint was marginal in all the three treatment groups, suggesting that umedaptanib pegol is effective to prevent the disease progression. The RAMEN study confirmed the cessation of disease progression. In the TEMPURA study, naïve nAMD patients showed improvement and no further macular degeneration, with striking improvement of visual acuity and central subfield thickness in some of the patients.

**Conclusions:**

These results demonstrate, for the first time, clinical proof of concept for aptamer based anti-FGF2 therapy of nAMD.

## Introduction

Age-related macular degeneration (AMD) is the leading cause of visual loss in individuals aged more than 50 years in developed countries [1]. In the United States, it is estimated that approximately 11 million individuals are affected by AMD, with a global prevalence of 170 million individuals [2]. The loss of central vision in exudative AMD (nAMD) is caused by choroidal neovascularization (CNV), due to vascular leakage and exudation, along with subretinal haemorrhage, which are often associated with subretinal fibrosis. Vascular endothelial growth factor (VEGF) plays a pivotal role in the pathogenesis of nAMD. When secreted by the retinal pigment epithelial (RPE) cells from the basal side, VEGF stimulates choroidal blood vessels and promotes expression of VEGF receptors on the inner choriocapillaris [3, 4]. Ultimately, these signaling networks lead to formation of new blood vessels that originate from the choroid, break through Bruch’s membrane, and infiltrate the neuro sensory retina [5].

The current standard therapy for nAMD is to target VEGF using ranibizumab (Lucentis^®^, Roche/Genentech), aflibercept (Eylea^®^, Regeneron Pharmaceuticals), bevacizumab (Avastin^®^, Roche/Genentech) and faricimab (Vabysmo^®^, bispecific anti-VEGF/anti-Ang2 drug, Roche/Genentech) although faricimab also inhibits Ang2 [4, 6]. All of them target the same molecule, VEGF. Frequent intravitreal injections of anti-VEGF drugs have been shown to be associated with major visual benefits in patients with AMD [7–9]. Nevertheless, the limitations of these therapies are well documented. In clinical trials, a significant portion of naïve (i.e., untreated) patients did not respond to anti-VEGF treatment [7–9]. Poor vision and persistent exudation are often associated with macular atrophy and submacular fibrotic scar formation despite different anti-VEGF treatments [10]. Clinical trials have also shown submacular fibrosis developing regardless of anti-VEGF treatment, causing further persistent vision loss in nAMD patients [11]. In addition, for patients frequent intravitreal injections are an additional major barrier to treatment compliance. There remains an obvious need for novel therapies with alternative mechanisms of action compared to anti-VEGF treatment for nAMD. It is noteworthy that poor or suboptimal response may be due to activity of other cytokines other than VEGF contributing to the neovascular process [12, 13].

Recent studies have shed light on the role of fibroblast growth factor-2 (FGF2) in disease progression of nAMD. FGF2 is a major member of the FGF family along with FGF1 and has been implicated in the pathophysiology of both angiogenesis and fibrosis through binding to tyrosine kinase FGF receptors, FGFR1-FGFR4 [14, 15]. FGF receptor double-conditional knockout (*Fgfr1/2*) mice showed a marked reduction in CNV accompanied by a decrease in the level of FGF2 upon laser injury [16]. FGF2 was the only essential ligand in the in vivo models of CNV, in keeping with FGF2 regulation of pathogenic angiogenesis via the STAT3 pathway [17]. Additionally, in the in vivo studies using mice and rats, anti-FGF2 aptamer, umedaptanib pegol (formerly called RBM-007), inhibits not only FGF2-induced angiogenesis but also laser-induced CNV, and CNV with fibrosis [14]. Pharmacokinetic studies of umedaptanib pegol in the rabbit vitreous revealed high and relatively long-lasting profiles [14]. Moreover, combined treatment with umedaptanib pegol and ranibizumab showed a synergistic effect in preventing CNV [14]. These findings strongly suggest that FGF2 should be a promising target molecule for nAMD therapy.

In the accompanying manuscript, we conducted the phase 1 (SUSHI) study to assess the safety, tolerability, and bioactivity of a single intravitreal injection of umedaptanib pegol in nine nAMD patients. Throughout these studies, umedaptanib pegol was safe, well tolerated, and reactive to nAMD patients that have persistent subretinal fluid refractory to anti-VEGF medications. Based on these results, phase 2 studies were conducted in anti-VEGF treated and treatment-naïve nAMD patients.

## Methods

### Investigational new drug, umedaptanib pegol

The drug substance for umedaptanib pegol intravitreal injection is a sodium salt of a single-stranded oligonucleotide aptamer with 37 structure-forming nucleotides in length that terminates at the 3′-end in an inverted 2′-deoxy-thymidine and at the 5′-end in an aminohexyl linker (see accompanying manuscript). Umedaptanib pegol binds strongly and specifically to FGF2 with the dissociation constant of 2 pM and blocks the interaction between human FGF2 and its receptors FGFR1-FGFR4 [14, 15]. In the accompanying phase 1 (SUSHI) study, three doses of 0.2, 1.0, or 2.0 mg were tested in the study eye and the top dose 2.0 mg was most effective, hence 2 mg was chosen throughout these studies.

### Phase 2 (TOFU) study design and participants

**Fig. 1:**
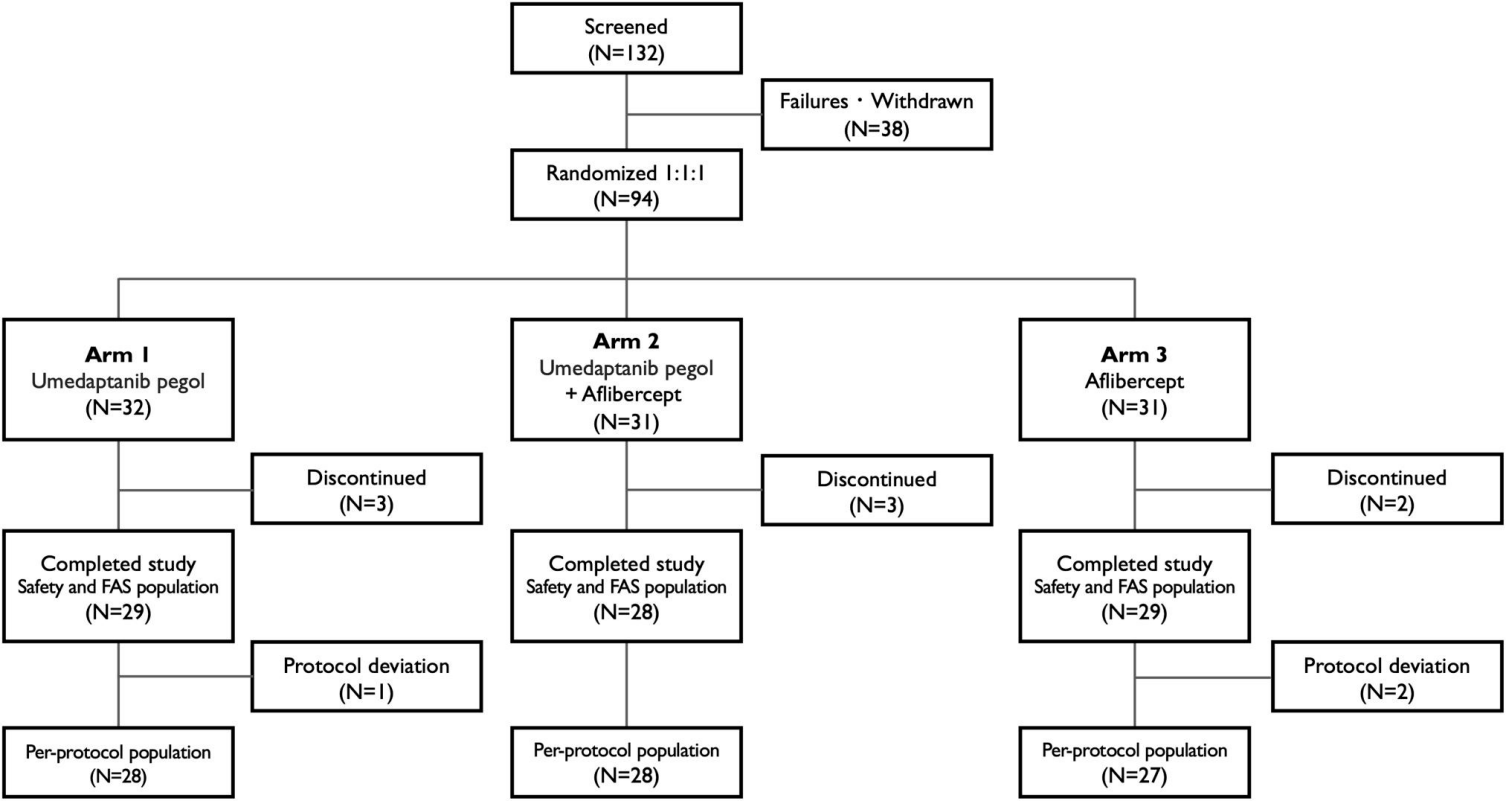
Trial profile. A total of 132 patients were screened and, after 38 withdrawals, a total of 94 patients were randomized to each of the treatment groups, Arm 1 (umedaptanib pegol monotherapy), Arm 2 (umedaptanib pegol + aflibercept combination therapy), and Arm 3 (aflibercept monotherapy), and subjected to clinical trials.

The study (www.clinicaltrials.gov identifier, NCT04200248) was conducted at eight study sites in the United States between December 2019 and December 2021. This is a multicentre, active-controlled, randomized and double masked study assessing the safety, efficacy and durability of four monthly intravitreal injections of umedaptanib pegol monotherapy in nAMD patients who were already on treatment with anti-VEGF therapy with poor/sub-optimal response to the treatment. In addition, the effects of four monthly umedaptanib pegol injections in combination with aflibercept dosed at every other month, compared to aflibercept monotherapy dosed at every other month were assessed. Eligible subjects were aged 55 years or older who had been diagnosed with active nAMD in the eye under study, for which previous standard treatment with intravitreal anti-VEGF agents (aflibercept, bevacizumab or ranibizumab) had demonstrated incomplete resolution of exudation, as assessed by spectral domain optical coherence tomography (SD-OCT). Other inclusion criteria were: best-corrected visual acuity (BCVA) of 78 to 24 letters (≤20/32 and ≥20/320 Snellen vision equivalent); presence of macular oedema or subretinal fluid on SD-OCT; absence of central atrophy or retinal pigmented epithelium tear in the fovea or any condition preventing visual acuity (VA) improvement in the eye under study. Subjects who met all eligibility criteria at baseline were randomized to receive treatment for up to three months. Subjects were randomized in a 1:1:1 ratio to either: Arm 1, Sham + umedaptanib pegol injectable solution (2.0 mg/eye); Arm 2, umedaptanib pegol injectable solution (2.0 mg/eye) + aflibercept; Arm 3, Sham + aflibercept (Fig. 1). Eligible subjects who were enrolled in the study were seen for at least 7 visits that included a Screening Visit (Visit 0), Baseline/Day 1 (Visit 1), and Weeks 1 to 20 (Visits 2–7). The primary analysis was conducted when all subjects completed the week 16 visit. The follow-up analysis was conducted at week 20 to assess the number of aflibercept injections used for rescue in addition to efficacy and safety data.

### Rescue therapy

Therapy considered necessary for the subject’s welfare that did not interfere with the evaluation of the study medication was allowed to be given at the discretion of the Principal Investigator. Rescue therapy is defined as intravitreal injection of aflibercept or other treatment at the discretion of investigator in the study eye. The rescue was to have been considered anytime during the study except for those visits with aflibercept injection as per protocol. The assessing physician should have determined the need for rescue and refer the subject to the injecting physician to decide about what to use for rescue. Eligibility criteria for rescue was as follow:

- BCVA decrease of >10 letters AND central subfield thickness (CST) increase of >50 μm from the last visit.
- The discretion of investigator.

If rescue was performed, the following assessments must have been performed within 30 min (except where indicated) after administration of rescue treatment (all ophthalmic procedures to be performed in the treated eye).

- Slit-lamp biomicroscopy
- Indirect ophthalmoscopy
- IOP: 40 (±10) minutes following rescue treatment

### Endpoints of the TOFU study

The primary endpoint was the mean change from baseline in BCVA at week 16, and secondary endpoints were the proportion of subjects with BCVA: (1) gain of ≥15 Early Treatment of Diabetic Retinopathy Study (ETDRS) letters (3-line gainers); (2) gain of ≥10 ETDRS letters; (3) gain of ≥5 ETDRS letters and (4) ≥15 letter loss from baseline at week 16. Central subfield thickness (CST), macular volume (MV), fibrosis, and SHRM were secondary efficacy variables, and change from baseline in CST, MV, fibrosis, and SHRM at week 16 were secondary endpoints.

### Phase 2 (RAMEN) study design and participants

This is a multi-center, open label, extension study (www.clinicaltrials.gov identifier, NCT04640272), in which 22 subjects who had exited the TOFU study received 4 monthly intravitreal injections of umedaptanib pegol (2.0 mg/eye). The primary endpoint of the study was at one month after the last injection with safety evaluation through two months post the last injection. For eligibility, subjects must have a confirmed diagnosis of nAMD in the study eye, for which previous TOFU masked treatment arms with intravitreal aflibercept and/or umedaptanib pegol has not demonstrated improvement in vision, with less than 15 letter BCVA improvement in TOFU study at the exit visit of TOFU over its baseline. Subjects must have completed all visits in the TOFU study. The primary endpoint was the mean change from baseline in BCVA at month 4. The secondary endpoint was the mean change from baseline in CST at month 4.

### Phase 2 (TEMPURA) study design and participants

**Fig. 2:**
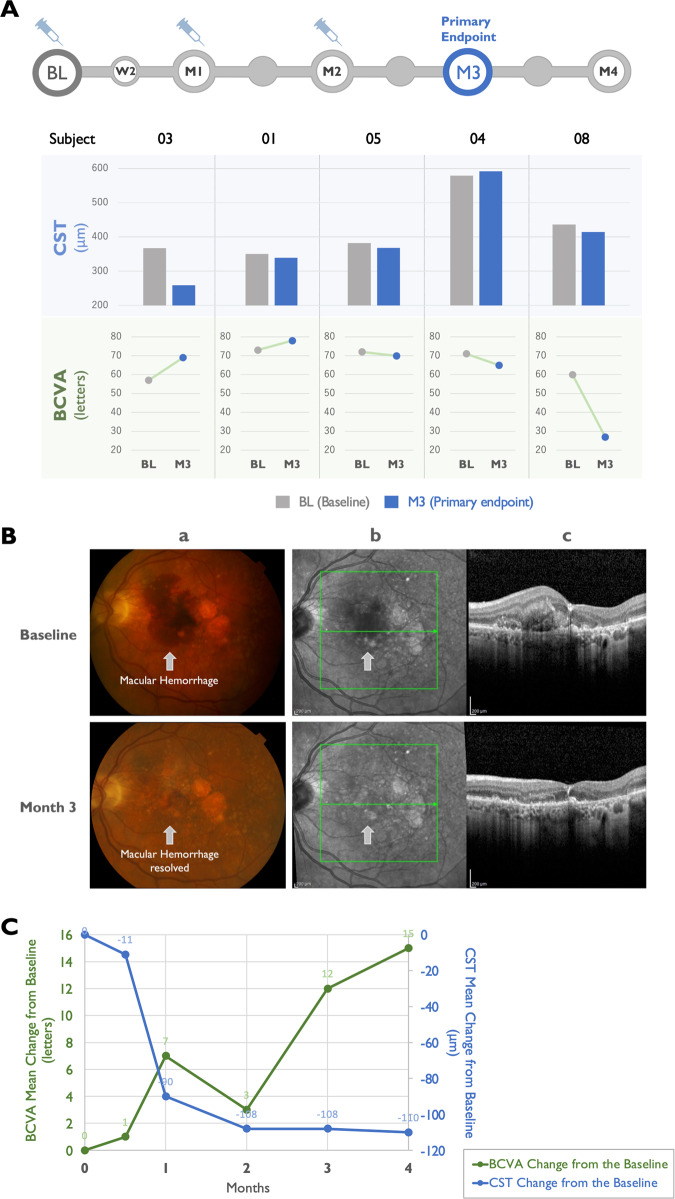
Effect of intravitreal umedaptanib pegol in treatment-naïve nAMD patients. **A** TEMPURA phase 2 study design and results. The top panel represents the study design. The lower panels present the baseline and primary endpoint data of CST and BCVA for subjects 03, 01, 05, 04 and 08. Note that CST and BCVA in subject 08 was not improved by rescue with aflibercept. **B** Dramatic drug effect in TEMPURA subject 03. **a** Fundus color picture. **b** Funds red-free picture. Extensive central macular haemorrhage (shown by arrows) at baseline (top panel) was quickly resolved after the first treatment with sustained improvement at primary endpoint month 3 (bottom panel). **c** Change of macular anatomy at central horizontal section in **b**. SD-OCT at baseline (top) and month 3 (bottom). **C** Coordinated change in vision (BCVA) and macular anatomy (CST) in TEMPURA subject 03. Changes of BCVA letters and CST are shown in green and blue, respectively.

The TEMPURA study (www.clinicaltrials.gov identifier, NCT04895293) was conducted as an investigator-sponsored trial (IST) at Midwest Eye Institute, Indiana, by Dr. Raj K Maturi between June 2021 and March 2022. This study is a single-center, open-label, 4-month study, designed to evaluate the safety and treatment efficacy of umedaptanib pegol in patients with untreated nAMD. Umedaptanib pegol treatment was performed at baseline, month 1, and month 2 visits with an intravitreal injection of 2.0 mg (100 μL) of umedaptanib pegol solution on the eye tested. All the subjects received treatment with the drug and those not responding were treated with rescue anti-VEGF drug, in addition to continuing umedaptanib pegol. Eligible subjects were 50 years or older and diagnosed with active nAMD. Other inclusion criteria were: BCVA of 5 to 73 letters (20/800-20/40 Snellen equivalent), inclusive, in the study eye; presence of CNV secondary to AMD; and clear ocular media and adequate pupil dilation to permit good quality photographic imaging. Five subjects who met all eligibility criteria at baseline were randomized to receive study medication of three monthly intravitreal injections of umedaptanib pegol (2 mg/eye) as shown in Fig. 2A (top panel). The primary and secondary endpoints were the mean change in CST and BCVA from baseline to 3 months, respectively.

### Clinical study sites

The TOFU study was conducted in the following clinical sites: Vitreo-Retinal Medical Group, Inc., Sacramento, CA (PI: Joel Pearlman), Bay Area Retina Associates, Walnut Creek, CA (PI: Subhransu Ray), Retinal Research Institute, LLC., Phoenix, AZ (PI: Mark R. Barakat), Medical Center Ophthalmology Associates, San Antonio, Texas (PI: Michael A. Singer), Valley Retina Institute, PA, McAllen, Texas (PI: Victor H. Gonzalez), Eye Institute, Indianapolis IN (PI: Raj K. Maturi), Georgia Retina, P.C., Marietta, GA (PI: Robert Stoltz) and Advanced Research LLC., Coral Springs, FL (PI: Shailesh Gupta).

### Drug administration procedure

Umedaptanib pegol for intravitreal injection is formulated in a proprietary, clear, aqueous solution. Intravitreal injections were given according to standard of care (SoC) techniques used in modern retinal practice. Briefly, a sterile lid speculum was placed, and local/topical anaesthesia was administered. The conjunctiva and ocular adnexa were prepared with povidone-iodine. A 30-gauge needle was used for all injections, which were given 4.0 mm from the limbus.

In the phase 2 (TOFU) study the IOP had to be ≤21 mmHg on the day of injection before the umedaptanib pegol injection could be administered. In patients in whom both intravitreal aflibercept and umedaptanib pegol were given (in the TOFU study), the injections were given consecutively (100 μl umedaptanib pegol first and then 50 μl aflibercept). In these eyes, the IOP was measured 40 (±10) minutes after the first injection was given, and if >21 mmHg, treated per the clinical investigator’s discretion and evaluated in 60 (±10) minutes. The second injection was performed just after IOP was <21 mmHg. The IOP was monitored after the second injection until it was <21 mmHg. If IOP persisted >21 mmHg and ≥10 mmHg increase from Baseline, it was reported as an AE.

### Randomization and blinding

Block randomization with randomly selected block sizes (3 or 6) is used. Only the study randomizer will know the true block sizes. Subjects who pass the screening and eligibility for this study will be allocated to Arm 1, 2 or 3 within each block according to the sequence of enrolment. The TOFU study is a double-masked study, with subjects and examiners including the principal investigator (PI) masked to the treatment regimen. Two arms in the TOFU study had sham injections which is a mimic procedure of a real intravitreal injection but do not penetrate the eye according to SoC techniques used in modern retinal practice. Briefly, the examination, and the injecting teams were masked so that the examiner was unaware of treatments received by study participants. The randomization and unblinding process followed Clindata Insight’s SOP-BM-06 (Rev. 1.0), dated 15 March 2018. All randomization requests and unblinding requests are sent by the sponsor to the designated representative of Clindata Insight and documented (FM-BM-06 Unblinding Request).

### Statistical methods and determination of sample size of TOFU study

The descriptive statistics included number of observations (n), mean, standard deviation, minimum, and maximum for continuous parameter and frequency (n) and percent (%) for categorical parameters. Details about the statistical analyses for this study were provided in the statistical analysis plan (SAP). This plan was created by an external statistician. All data manipulations and descriptive summaries were implemented using SAS® Statistical Analysis Software Version 9.4 or higher. The study was not powered for statistical significance.

### Ethics statement

The clinical studies were conducted at 8 (TOFU and RAMEN) and 1 (TEMPURA) study sites in compliance with the Declaration of Helsinki, US Code 21 of Federal Regulations, and the Harmonized Tripartite Guidelines for Good Clinical Practice (1996); and were reviewed and approved by the appropriate Ethics Committees or institutional review boards (Advarra, single IRB used, 100 Merriweather Dr., Suite 600, Columbia, MD 21044). Informed consent was obtained from all study participants. Patients were not compensated for trial participation.

## Results

### TOFU study

A total of 86 patients were randomized to each of the treatment groups as follows: umedaptanib pegol monotherapy 100 μL (2.0 mg/eye) (Arm 1, *n* = 29), umedaptanib pegol 100 μL (2.0 mg/eye) + aflibercept 50 μL (2.0 mg/eye) combination therapy (Arm 2, *n* = 28), and aflibercept monotherapy 50 μL (2.0 mg/eye) (Arm 3, *n* = 29). The trial profile is shown in Fig. 1. Baseline demographic features were balanced between treatment groups although the Arm 1 population was biased toward aged patients with a longer history of treatment (Table 1). Subject baseline characteristics in this study were broadly consistent with the general characteristics of patients with nAMD. Most patients (98%) were Caucasian, the mean participant age was 78 years, and the mean baseline BCVA was 63 ETDRS letters. It is important to note that while our study targeted patients with poor/sub-optimal response to anti-VEGF treatments, selection and enrolment criteria were based upon anatomical measures rather than visual acuity, in which previous treatment with anti-VEGF agents (at least 4 injections over the past 8 months) had demonstrated incomplete resolution of exudation, as assessed by OCT. Subjects were eligible for rescue with aflibercept or other treatment at the discretion of the investigator anytime during the study except for those receiving aflibercept injections as per protocol (summarized in Supplementary Table 1).

**Table 1:** Baseline demographics of TOFU study.

Variables	Arm 1 (*N* = 29)	Arm 2 (*N* = 28)	Arm 3 (*N* = 29)	Total (*N* = 86)
Age (years)				
Mean (SD)	80.41 (7.23)	75.07 (7.76)	77.72 (6.57)	77.77 (7.44)
Age Group (years)				
55 to 64 Years	1 (3.4%)	2 (7.1%)	0	3 (3.5%)
65 to 74 Years	5 (17.2%)	11 (39.3%)	13 (44.8%)	29 (33.7%)
75 to 84 Years	14 (48.3%)	12 (42.9%)	10 (34.5%)	36 (41.9%)
≧85 Years	9 (31.0%)	3 (10.7%)	6 (20.7%)	18 (20.9%)
Gender				
Male	11 (37.9%)	14 (50.0%)	13 (44.8%)	38 (44.2%)
Female	18 (62.1%)	14 (50.0%)	16 (55.2%)	48 (55.8%)
Race				
White	27 (93.1%)	28 (100%)	29 (100%)	84 (97.7%)
Asian	1 (3.4%)	0	0	1 (1.2%)
Native Hawaiian or Other Pacific Islander	1 (3.4%)	0	0	1 (1.2%)
Ethnicity				
Hispanic or Latino	2 (6.9%)	1 (3.6%)	2 (6.9%)	5 (5.8%)
Not Hispanic or Latino	27 (93.1%)	27 (96.4%)	27 (93.1%)	81 (94.2%)
BCVA (No. of Letters)				
Mean (SD)	60.24 (14.00)	66.71 (10.81)	61.52 (13.83)	62.78 (13.13)
BCVA Category				
<55 letters	9 (31.0%)	2 (7.1%)	8 (27.6%)	19 (22.1%)
≧55 letters	20 (69.0%)	26 (92.9%)	21 (72.4%)	67 (77.9%)
Central Subfield Thickness (µm)				
Mean (SD)	452.07 (138.24)	398.04 (124.01)	437.55 (129.34)	429.58 (131.20)
Central Subfield Thickness Category				
<400 µm	11 (37.9%)	15 (53.6%)	12 (41.4%)	38 (44.2%)
≧400 µm	18 (62.1%)	13 (46.4%)	17 (58.6%)	48 (55.8%)
Duration of Diagnosis (years)				
Mean (SD)	4.31 (2.91)	4.60 (4.28)	3.63 (4.31)	4.17 (3.86)
Duration of Diagnosis Categories				
≦1 Year	3 (10.3%)	7 (25.0%)	7 (24.1%)	17 (19.8%)
1–5 Years	15 (51.7%)	9 (32.1%)	15 (51.7%)	39 (45.3%)
>5 Years	11 (37.9%)	12 (42.9%)	7 (24.1%)	30 (34.9%)

Primary and secondary endpoint data of mean change in BCVA and CST from baseline to 16 weeks are summarized in Table 2. Three patients (one in Arm1 and two in Arm 3) were excluded from the Full Analysis Set (FAS) due to protocol deviations, providing the Per-Protocol (PP) dataset. The bioactivity of umedaptanib pegol was confirmed since most of the subjects in the umedaptanib pegol monotherapy group remained stable both for BCVA and CST measurements during the study. Notably, the change in BCVA and CST at week 16 (primary endpoint) was very marginal in all three treatment groups, suggesting that umedaptanib pegol is effective in preventing disease progression. The anti-VEGF pretreated study population appeared to be less sensitive not only to anti-VEGF but also to umedaptanib pegol. Under these conditions, umedaptanib pegol did not show superiority, either as monotherapy or in association with aflibercept, compared to aflibercept monotherapy in this previously treated nAMD population (Table 2). Scanning individual OCT images revealed that umedaptanib pegol did not show an anti-fibrotic effect on pre-existing fibrosis in the TOFU subjects with chronic nAMD.

**Table 2:** Mean changes in BCVA (a) and CST (b) from baseline to week 16 in the TOFU study.

Visit		Arm 1 (*N* = 28)	Arm 2 (*N* = 28)	Arm 3 (*N* = 27)
(a) Mean change in BCVA				
BL	LS Mean (SE)	60.5 (2.43)	66.7 (2.43)	62.6 (2.47)
W16	LS Mean (SE)	53.9 (3.32)	65.1 (2.79)	65.0 (2.90)
	LS Mean Change from BL (SE)	−4.8 (2.17)	−1.2 (1.82)	2.2 (1.86)
	*p*-value	0.016	0.188	
(b) Mean change in CST				
BL	LS Mean (SE)	451.0 (24.83)	398.0 (24.83)	446.3 (25.28)
W16	LS Mean (SE)	486.0 (29.58)	395.1 (24.81)	409.1 (25.79)
	LS Mean Change from BL (SE)	37.1 (15.18)	−6.1 (12.75)	−19.4 (13.15)
	p-value	0.006	0.472	

As mentioned above, the Arm 1 population was biased towards a longer history of treatment (Table 1). To examine the possible effect of umedaptanib pegol on patients with a relatively shorter history of treatment, a post-hoc supportive analysis was conducted by collecting patients with a treatment history of less than 2 years, comprising 5, 7, and 12 patients in Arm 1, Arm 2 and Arm 3, respectively. There appears to be no significant difference in BCVA change in Arm 1 and Arm 3, and Arm 2 also, although the collected patient size is limiting (Supplementary Fig. 1), suggesting similar action of umedaptanib pegol to aflibercept in nAMD patients of short or no treatment history.

In this study, the most common ocular AEs were consistent with those typically seen with the intravitreal injection procedure and in a population of patients with nAMD (Table 3). The ocular safety profiles of umedaptanib pegol alone and the combination therapy were consistent with that of the anti-VEGF SoC in previously reported studies. There were no unexpected ocular AEs such as retinal vasculitis or occlusive events. No drug related systemic AEs were reported. In this study, (serious) AEs that emerged after ocular treatment were endophthalmitis (infectious), iritis, vitritis, vision blurred, and eye pain. Iritis/vitritis and its associated symptoms (vision blurred, eye pain) were reported as possibly related to umedaptanib pegol (Supplementary Table 2). These events resolved quickly with topical treatment showing good outcomes with complete recovery without sequelae.

**Table 3:** Overall summary of treatment emergent adverse events (TEAEs) in the TOFU study.

	Arm 1			Arm 2			Arm 3		
	(*N* = 28)			(*N* = 29)			(*N* = 29)		
	*n*	%	E	*n*	%	E	*n*	%	E
Subjects with at least one TEAE	21	75	67	21	72.4	47	15	51.7	38
Subjects with at least one Ocular TEAE	16	57.1	41	19	65.5	38	10	34.5	19
- Study Eye	15	53.6	36	17	58.6	34	8	27.6	14
- Non-study Eye	8	28.6	13	5	17.2	7	6	20.7	8
Subjects with at least one Non-ocular TEAE	13	46.4	26	6	20.7	9	9	31	19
Subjects with at least one TEAE Related to Study Drug	3	10.7	9	2	6.9	8	0		0
Subjects with at least one Procedure TEAE Related to Injection	9	32.1	15	10	34.5	14	6	20.7	9
Subjects with at least one serious TEAE	3	10.7	6	2	6.9	4	3	10.3	3
Subjects with at least one serious TEAE related to Study Drug	2	7.1	5	1	3.4	3	0		0
Subjects with at least one serious TEAE related to Injection Procedure	0		0	1	3.4	1	0		0

One subject in the Sham + umedaptanib pegol group (Arm 1) received umedaptanib pegol+aflibercept at baseline visit by rescue treatment. This subject was included in the umedaptanib pegol+flibercept group (Arm 2) for all safety analysis.

Note. A treatment-emergent adverse event (TEAE) is defined as an adverse event that occurred or worsened following the first administration of the study drug. If a subject has multiple occurrences of a TEAE, the subject is presented only once in the subject count(n) column. Occurrences are counted each time in the occurrence/event(E) column. Adverse events were coded using MedDRA Ver 23.0.

### RAMEN study

Additional intravitreal injections of umedaptanib pegol (2.0 mg) were shown to be safe. There were no drug related treatment-emergent adverse events (TEAEs) during the RAMEN study (Supplementary Table 3). The most common ocular AEs were related to the injection procedure and were consistent with those typically seen with intravitreal injections in a population of patients with nAMD. There were no drug related systemic AEs reported in the study.

During the monthly intravitreal injections of umedaptanib pegol, the BCVA and CST scores stayed unchanged or slightly worsened if any. Considering the typical outcome of nAMD subjects without treatment, this relative stability suggests biological activity of umedaptanib pegol to halt the disease progression. Of 20 rolled-over subjects excluding two who missed scheduled injections, seven came from Arm 1 (umedaptanib pegol monotherapy), five from Arm 2 (umedaptanib pegol/aflibercept combination therapy) and eight from Arm 3 (aflibercept monotherapy). Interestingly, the change in BCVA and CST showed distinct response depending on each Arm (Supplementary Fig. 2). The vison (BCVA) stayed better in Arm 1 and Arm 2 than in Arm 3, and the macular anatomy (CST) became less worsened in Arm 2 than in Arm 1 and Arm 3. The meaning of these observations is not immediately obvious but seems to reflect different modes of action between umedaptanib pegol and aflibercept (discussed later).

Examination of serial OCT images revealed that umedaptanib pegol did not have any effect on severity of pre-existing fibrosis in the RAMEN subjects with chronic nAMD. Specifically, the pre-existing fibrosis remained stable without worsening.

### TEMPURA study

There were 5 scheduled visits during the study and the assessments for each visit are described in Fig. 2A (top panel). Five subjects participated in the study, and from those, 3 subjects showed indication of a positive effect of umedaptanib pegol; one of these, subject 03, showed dramatic improvement after the first injection of umedaptanib pegol (Fig. 2A). No drug-related AEs were reported during the study; 2 AEs (1 positive COVID test, 1 subretinal haemorrhage) were reported and resolved without sequelae; 2 non-ocular serious AEs (1 minor heart attack, 1 severe diarrhoea) occurred, and both were assessed as not related to the study medication and resolved with appropriate treatment. Detailed information on the outcomes for each one of the 5 subjects included in the TEMPURA study is described below.

#### SUBJECT 01 (77 years old male, Caucasian)

In this case, the subject presented with mild subretinal fluid at the baseline visit. After umedaptanib pegol therapy, improvement in both vision (BCVA) from 73 letters on the baseline to 78 letters on month 3 and CST from 350 µm on the baseline to 339 µm on month 3 was seen (Fig. 2A subject 01).

#### SUBJECT 03 (66 years old male, Caucasian)

In this case, the subject presented with a large subretinal haemorrhage associated with an active CNV at the baseline visit (Fig. 2A subject 03). After umedaptanib pegol therapy there was an improvement in both vision (BCVA) from 57 letters on the baseline to 69 letters on month 3 and CST from 367 µm on the baseline to 259 µm on month 3 (Fig. 2A subject 03, Fig. 2Bc). Moreover, extensive central macular haemorrhage at baseline quickly resolved after the first treatment, with sustained improvement at month 3 (Fig. 2Bab) without obvious subretinal fibrosis. The improvements of vision (BCVA) and CST were correlated in this subject during the treatment (Fig. 2C).

#### SUBJECT 04 (94 years old female, Caucasian)

In this case, the subject presented with CNV and a pigment epithelial detachment (PED) at the baseline visit. There was an initial BCVA improvement from 71 letters on the baseline to 74 letters on month 2 after the first study treatment with umedaptanib pegol. This subject missed the month 1 visit due to hospitalization for a not drug related severe AE (minor heart attack), missing one dose of umedaptanib pegol per-protocol doses. The subject returned to the study on month 2. Despite the treatment, BCVA dropped to 65 letters on month 3. On SD-OCT the macular fluid persisted, and CST went from 579 µm on the baseline to 559 µm on month 2 and then 592 µm on month 3. This subject received rescue treatment with anti-VEGF (aflibercept) on month 4 (2 months without treatment) with BCVA 60 letters and CST 648 µm (Fig. 2A subject 04).

#### SUBJECT 05 (82 years old female, Caucasian)

In this case, the subject presented with active CNV associated with subretinal fluid, showing a slight improvement on OCT not translated to BCVA during the study. BCVA was 72 letters on the baseline and dropped to 70 letters on month 3. CST was 382 µm on baseline decreasing to 368 µm on month 3 (Fig. 2A subject 05).

#### SUBJECT 08 (74 years old female, Caucasian)

In this case, the subject presented with active CNV inferior to the fovea that quickly developed subretinal fibrosis despite the treatment with umedaptanib pegol and two rescue treatment with aflibercept between month 1 and month 2 and on the month 4 visit. Outcomes for vision (BCVA) and CST were as follows: BCVA from 60 letters on the baseline to 27 letters on month 3 and CST from 436 µm on the baseline to 414 µm on month 3. Visual acuity loss was not stopped by umedaptanib pegol nor aflibercept. This subject had a severe AE not related to the study (severe diarrhoea) that resolved without sequelae and did not influence the study visits (Fig. 2A subject 08).

The results across the five subjects were heterogenous; this is to be expected even with SoC treatment for nAMD, especially in the initial 3–4 months of treatment, when in clinical experience shows that variable responses to treatment are typical. These findings suggest that umedaptanib pegol can be effective in improving vision and retinal anatomy in treatment-naïve nAMD patients.

## Discussion

A significant unmet need exists with anti-VEGF monotherapy regardless of the benefit in anti-VEGF medications in patients with nAMD [18–24]. Numerous clinical trials have been completed or are underway to fill the remaining unmet needs of the anti-VEGFs. However, the vitreoretinal community (and patients) have been disappointed with repeated failures of highly publicized drugs—most notably the anti-platelet-derived growth factor (anti-PDGF, Fovista^®^: Ophthotech, New York, NY) [25]. PDGF binds to a tyrosine kinase receptor on pericytes, which likely protects endothelial cells from anti-VEGF drugs [26]. Despite the expectation that anti-PDGF could enhance the anti-VEGF action by targeting pericytes, the phase 3 clinical trials that enrolled 1248 patients have shown no vision or anatomic advantages of anti-PDGF in combination therapy compared with monotherapy of anti-VEGFs [25–27]. While previous studies with anti-Ang2 drugs (nesvacumab, Regeneron) showed an effect but there was no additional benefit in the combination [28], a bispecific anti-VEGF/anti-Ang2 drug (faricimab, Roche) was recently approved [29, 30].

Clinical studies presented here are the first clinical trials to target FGF2 via intravitreal monotherapy or combination therapy with anti-VEGF in pretreated nAMD patients (TOFU study) and monotherapy in treatment-naïve patients with nAMD (TEMPURA study). The TOFU study did not show any improvement with umedaptanib pegol in patients, who had previously undergone anti-VEGF treatment, however, it did prevent disease progression. On the other hand, most of the subjects in the TEMPURA study showed improvement or stable control in BCVA/CST, and a pronounced effect was demonstrated in a few treatment-naïve subjects. That one subject showed complete resolution of macular fluid and even subretinal haemorrhage, and this improvement was rapid and sustained, pointing to a clear beneficial effect of umedaptanib pegol. Moreover, the post-hoc analysis of the TOFU study suggested that umedaptanib pegol is effective and non-inferior to aflibercept in nAMD patients with a short history of anti-VEGF treatment although the collected patient size is limiting (Supplementary Fig. 1). These findings provide a clinical proof of concept for this first example of anti-FGF2 therapy in nAMD, demonstrating that FGF2 plays a pivotal role in nAMD disease progression.

Although umedaptanib did not show superiority, either as monotherapy (Arm 1) or in association with aflibercept (Arm 2), compared to aflibercept monotherapy (Arm 3) in the TOFU study, it is noteworthy that the Arm 1 population was biased not only towards aged patients with a longer history of treatment but also towards patients with a relatively advanced disease stage. The population of patients with baseline BCVA of less than 65 in the PP dataset was 61% (17/28), 36% (10/28), and 44% (12/27) in Arm 1, Arm 2, and Arm 3, respectively (Table 1). Therefore, it is reasonable to suspect that the Arm 1 population is less responsive to medications. Another interesting speculation is plausible from the RAMEN study. Deterioration in visual acuity is most pronounced in Arm 3 population compared to Arm 1 and Arm 2 populations during the extended treatment with umedaptanib pegol (Supplementary Fig. 2). Therefore, we assume that conversion of patients previously treated with anti-VEGF to umedaptanib pegol results in subsequent deterioration of visual acuity. This might explain, at least in part, the result that Arm 1 (umedaptanib pegol monotherapy) showed the least improvement in visual acuity in the TOFU study (Table 2). Consistently, rescue populations upon worsening in visual acuity are higher in Arm 1 than in Arm 2 and 3 (Supplementary Table 1), suggesting that switching SoC patients to a different-acting umedaptanib pegol treatment appears to cause vision loss. Given these considerations, further clinical trials of umedaptanib pegol are inevitable in treatment-naïve nAMD.

To the best of our knowledge, this is the first clinical evidence that a non-VEGF target can lead to the successful monotherapy of nAMD. Most clinical trials carried out or underway in the past decade involved modifications of the dose, formulation, or administration regimen of anti-VEGF medications. Anti-FGF2 therapy should provide a novel approach to treat nAMD with a distinct mechanism of action. The present studies strongly suggest that umedaptanib pegol must be a first-line medication before anti-VEGF treatment. The efficacy of umedaptanib pegol in combination with anti-VEGFs and the efficacy of anti-VEGF administration after umedaptanib pegol treatment needs to be investigated in naïve nAMD. The half-life of umedaptanib pegol in the vitreous liquid is substantially longer than approved drugs such as ranibizumab and aflibercept [14]. We plan to organize larger controlled phase 3 studies of umedaptanib pegol in treatment of naïve nAMD with an interdose interval longer than one month. Furthermore, as we show, umedaptanib pegol holds promise as an additive therapy to anti-VEGF treatments for nAMD by blocking subretinal fibrosis.

Finally, it can be emphasized that aptamer is a good modality to treat macular diseases as shown first by the approved anti-VEGF aptamer (Macugen^®^, Eyetech Pharmaceuticals, New York, NY) [31] in nAMD and as evidenced by this study and the recent development of anti-C5 aptamer (Zimura^®^, IVERIC bio Inc, New York, NY) [32] in dry AMD. Aptamer holds several pharmaceutical advantages such as capturing target shape, middle molecule, chemical synthesis, production cost, and low antigenicity, thus providing many unmet opportunities for aptamer-based therapeutics in ophthalmology.

## Summary

### What was known before

- Intravitreal anti-VEGF drugs (e.g., bevacizumab, ranibizumab, and aflibercept) have become the standard treatment for nAMD.
- As far as is known, VEGF is the only effective target molecule for nAMD monotherapy.
- Participants may require a high injection frequency over years of treatment, leading to a high treatment burden or several complications.
- The real-world studies showed worse visual outcomes, possibly due to poor compliance.

### What this study adds

- Intravitreal umedaptanib pegol was safe, well tolerated, and effective to treat naïve nAMD patients.
- This report demonstrates clinical proof of concept for anti-FGF2 therapy in nAMD.
- To the best of our knowledge, this is the first demonstration of nAMD therapy with targets other than VEGF.

### Supplementary information

Supplementary Material

## Data Availability

The datasets used and/or analysed during the current study are available from the corresponding author on reasonable request.
