# Supplementary material for: Clinical proof of concept for anti-FGF2 therapy in exudative age-related macular degeneration (nAMD): phase 2 trials in treatment-naïve and anti-VEGF pretreated patients
Source: Eye (Lond). 2023 Nov 30;38(6):1140–8. doi: 10.1038/s41433-023-02848-7 (PMC11009322; doi:10.1038/s41433-023-02848-7)
Supplement: Supplementary file 1 — Supplementary Material [file 41433_2023_2848_MOESM1_ESM.pdf]

SUPPLEMENTARY MATERIALS

Supplementary Fig. 1. Post-hoc analysis of the TOFU dataset selecting a short history of treatment of less than 2 years. Bar graph showing the primary efficacy end point, mean change in visual acuity (VA) from baseline to week 16. Data from the post-hoc analysis is depicted. Error bars represent standard error.

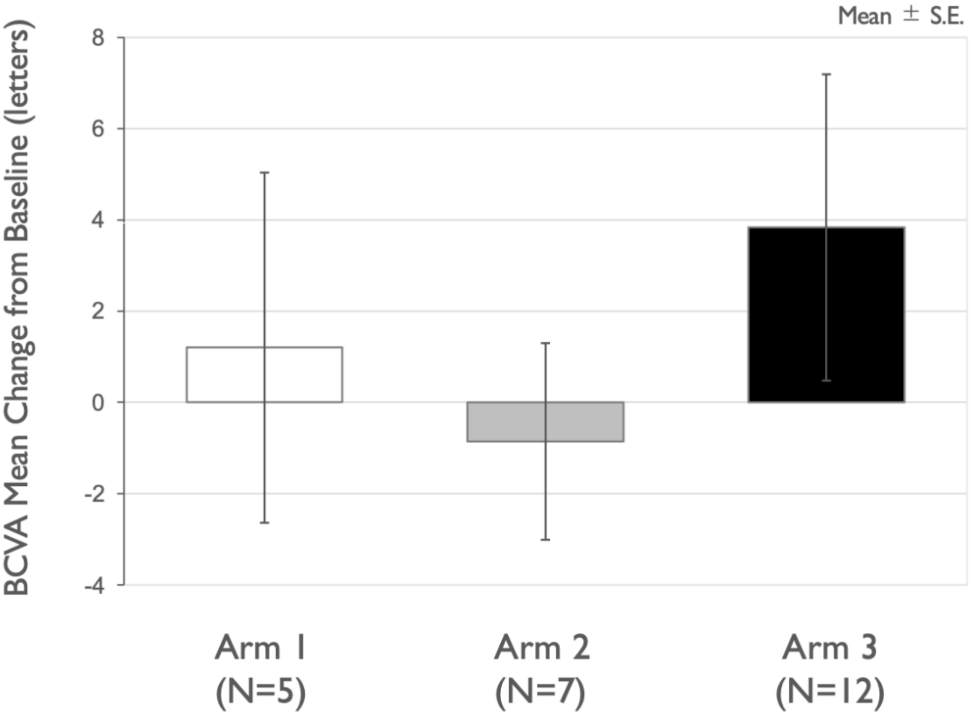

Supplementary Fig. 2. The primary and secondary endpoint dataset in the RAMEN study. Changes in BCVA (vision, **a**) and CST (macular anatomy, **b**) during extended UMEDAPTANIB PEGOL injections in each Arm populations are shown. Twenty subjects were rolled-over from Arm 1 (seven subjects), Arm 2 (five subjects) and Arm3 (eight subjects) and received four monthly intravitreal injections of UMEDAPTANIB PEGOL. Error bars represent standard errors.

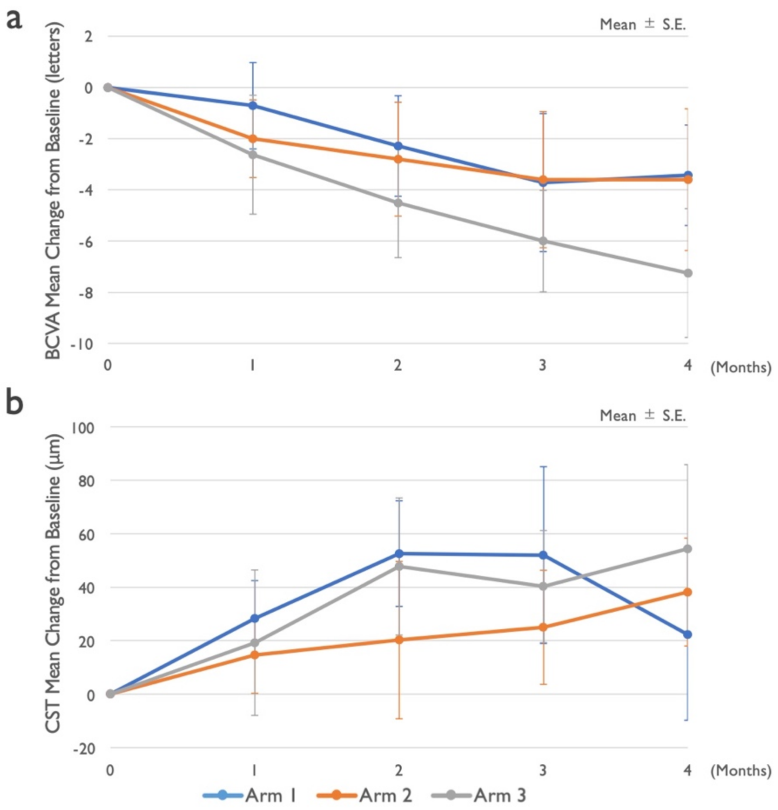

Supplementary Table 1. Number of subjects who met rescue therapy criteria by visit in the TOFU study (Full Analysis Set).

	Arm 1 (N=29)		Arm 2 (N=28)		Arm 3 (N=29)	
Visit	n	%	n	%	n	%
Week 1	0	0	0	0	0	0
Week 4	0	0	0	0	1	3.4
Week 8	2	6.9	0	0	2	7.1
Week 12	7	24.1	0	0	0	0
Week 16	3	10.3	5	17.9	4	14.3
Week 20	16	55.2	5	17.9	8	29.6

Supplementary Table 2. Summary of ocular treatment emergent serious adverse events (TESAEs) in the TOFU study.

	Arm 1 (N=28)		Arm 2 (N=29)		Arm 3 (N=29)	
	n	%	n	%	n	%
Subjects with at least one ocular TESAE	2	7.1	2	6.9	1	3.4
Eye disorders	2	7.1	1	3.4	1	3.4
- Iritis	2	7.1	1	3.4	0	0
- Vitritis	2	7.1	1	3.4	0	0
- Eye pain	1	3.6	0	0	0	0
- Neovascular age-related macular degeneration	0	0	0	0	1	3.4
- Vision blurred	0	0	1	3.4	0	0
Infections and infestations						
- Endophthalmitis	0	0	1	3.4	0	0

Note: A treatment-emergent adverse event (TEAE) is defined as an adverse event that occurred or worsened following the first administration of the study drug. If a subject has multiple occurrences of a serious TEAE, the subject is presented only once in the subject count(n) column.

Supplementary Table 3. Summary of ocular treatment emergent adverse events (TEAEs) in the RAMEN study.

	RAMEN (N=22)		
	n	%	E
Subjects with at least one TEAE	6	27.3	19
Subjects with at least one Ocular TEAE	5	22.7	8
- Study Eye	4	18.2	7
- Non-study Eye	1	4.5	1
Subjects with at least one Non-ocular TEAE	2	9.1	11
Subjects with at least one TEAE Related to Study Drug	0		0
Subjects with at least one TEAE Related to Injection Procedure	2	9.1	5
Subjects with at least one serious TEAE	1	4.5	1
Subjects with at least one serious TEAE related to Study Drug	0		0
Subjects with at least one serious TEAE related to Injection Procedure	0		0
Subjects with a TEAE leading to premature discontinuation	0		0
Subjects with a TEAE leading to death	0		0
